# Effect of age and sex on immune checkpoint expression and kinetics in human T cells

**DOI:** 10.1186/s12979-020-00203-y

**Published:** 2020-11-04

**Authors:** Rosanne D. Reitsema, Rebeca Hid Cadena, Sander H. Nijhof, Wayel H. Abdulahad, Minke G. Huitema, Davy Paap, Elisabeth Brouwer, Annemieke M. H. Boots, Peter Heeringa

**Affiliations:** 1 Department of Rheumatology and Clinical Immunology, University of Groningen, University Medical Center Groningen, Groningen, The Netherlands; 2 Department of Pathology and Medical Biology, University of Groningen, University Medical Center Groningen, Groningen, The Netherlands

**Keywords:** Immune checkpoints, Age, Sex, T cells, PD-1, CD40L, VISTA, CD28

## Abstract

**Background:**

Immune checkpoints are crucial molecules in maintaining a proper immune balance. Even though age and sex are known to have effects on the immune system, the interplay between age, sex and immune checkpoint expression by T cells is not known. The aim of this study was to determine whether age and sex affect immune checkpoint expression by T cells and if age and sex affect the kinetics of immune checkpoint expression following *ex vivo* stimulation. In this study, whole blood samples of 20 healthy young adults (YA, 9 males and 11 females) and 20 healthy older adults (OA, 9 males and 11 females) were stained for lymphocyte lineage markers and immune checkpoints and frequencies of CD28+, PD-1+, VISTA+ and CD40L+ T cells were determined. Immune checkpoint expression kinetics were studied following *ex vivo* anti-CD3/anti-CD28 stimulation of T cells from young and older healthy adults.

**Results:**

We report an age-associated increase of CD40L + CD4+ and CD40L + CD8+ T-cell frequencies, whereas CD40+ B-cell frequencies were decreased in older adults, suggesting modulation of the CD40L-CD40 interaction with age. Immune checkpoint expression kinetics revealed differences in magnitude between CD4+ and CD8+ T cells independent of age and sex. Further analysis of CD4+ T-cell subsets revealed an age-associated decrease of especially PD-1 + CD4+ memory T cells which tracked with the female sex.

**Conclusion:**

Collectively, our results demonstrate that both age and sex modulate expression of immune checkpoints by human T cells. These findings may have implications for optimising vaccination and immune checkpoint immunotherapy and move the field towards precision medicine in the management of older patient groups.

**Supplementary Information:**

The online version contains supplementary material available at 10.1186/s12979-020-00203-y.

## Background

Age and sex are associated with many changes in immune function and with development of multiple (auto) inflammatory diseases such as rheumatoid arthritis, systemic lupus erythematosus (SLE), giant cell arteritis and polymyalgia rheumatica [1–3]. Major age-associated changes in the immune system include a decrease in the number of lymphocytes, especially naive CD8+ T cells, and a decrease in the diversity of the T-cell receptor repertoire [4, 5]. In addition, humoral immunity wanes with ageing, which is presumably caused by both decreases in B cells and in the production of high-affinity antibodies [6–8]. Compared to age-related changes, the effect of sex on immune function is less well understood. Nevertheless, it is known that there are multiple differences in the immune system between males and females. In general, females show stronger immune responses, including stronger T-cell responses, which may lead to an increased protection against different pathogens [9, 10]. In addition, female sex hormones such as estrogen enhance B-cell responses [11]. Consequently, a more active immune system in females during the reproductive years might render females prone to the development of inflammation and autoimmune related conditions [12].

Immune checkpoints (ICs) are pivotal molecules in the regulation of the immune response and thus important when studying age- and sex-associated effects on the immune system. Several IC molecules are currently targeted to treat cancer and chronic infectious diseases. During chronic infection and cancer, T cells become exhausted, a state of poor effector function, which can be reversed by immunotherapy via the antagonistic targeting of inhibitory ICs [13]. Unfortunately, by activating the immune system to boost the immune response to tumour cells, several immune-related adverse events (irAEs) affecting multiple organs of the body can occur [14]. Among these irAEs, development of rheumatic diseases has been reported [15, 16], which underlines the importance of ICs in inflammatory diseases and adds to the complexity of IC therapy.

The best studied ICs belong to the so called B7 family, which consists of the co-stimulatory IC CD28 and the co-inhibitory ICs programmed death 1 (PD-1) and the more recently described V-domain Ig suppressor of T-cell activation (VISTA) [17, 18]. Regarding ageing effects, several studies report on decreased CD28 expression by CD8+ and to a lesser extent CD4+ cells of older adults, whereas limited data is available on effects of age and sex on the expression of other ICs [4, 19, 20].

The aim of this study was to determine whether age and sex affect IC expression by T cells and if age and sex affect the kinetics of IC expression following *ex vivo* stimulation. To this end, we investigated expression and kinetics of the co-stimulatory molecules CD28 and CD40L and the co-inhibitory molecules PD-1 and VISTA on both CD4+ and CD8+ cells in young and older males and females. In addition, we investigated IC expression by naive and memory CD4+ T-cell subsets and CD40 expression by B cells. Age- and sex- dependent differences in IC expression may underlie the higher propensity of females to develop inflammation and autoimmune conditions. Furthermore, the knowledge obtained could be important for optimising current vaccination and immunotherapies for the ageing world populations and aid the development of precision medicine.

## Results

### Effects of age and sex on numbers of circulating immune cells

**Fig. 1:**
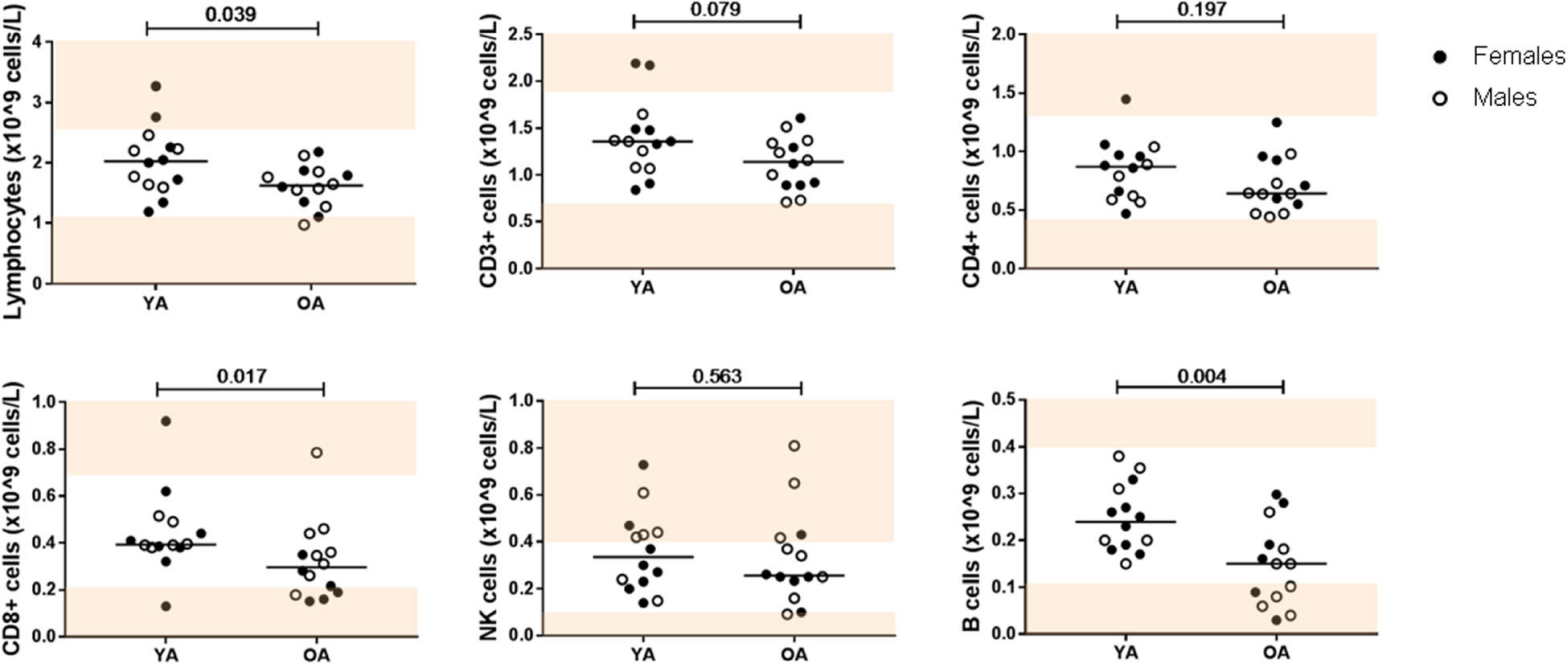
Absolute cell counts in peripheral blood of young and older healthy adults. Absolute cell counts of total lymphocytes, CD3+, CD4+ and CD8+ T cells, NK cells and B cells were determined by TruCount. Horizontal bars reflect median values. Light pink areas represent values outside the reference range. Reference values were provided by the department of Laboratory Medicine (UMCG). Solid circles represent females and open circles represent males. The Mann-Whitney U test was used for comparisons between young (YA, *n* = 14) and older adults (OA, n = 14). *P*-values are indicated in the graphs

As ageing has been associated with alterations in peripheral blood immune cell counts, we first determined absolute leukocyte counts in peripheral blood samples of healthy young adults (YA, *n* = 14) and older adults (OA, n = 14) by TruCount (Fig. 1, Additional Table S1). We confirmed decreases of total lymphocyte numbers in OA (*p* = 0.039). Furthermore, total T-cell (CD3+) numbers tended to be decreased in OA (*p* = 0.079) which was mainly due to decreases in CD8+ (*p* = 0.017) but not CD4+ cells. In addition, B-cell numbers were decreased in OA (*p* = 0.004). No statistical differences in immune cell numbers were detected between males and females (Additional Fig. S1).

Next, whole blood staining of CD4+ cells in 20 YA and 20 OA employing CD4, CD45RA and CD25 revealed increased ratios of memory over naive T cells in the older group, as OA had a higher frequency of memory CD4+ cells (CD45RA-) than YA (*p* = 0.028) (Additional Fig. S2A). Of note, the selected markers (CD45RA and CD25) chosen to analyse naive and memory CD4+ T-cell subsets according to the Miyara classification [21], do not allow an accurate analysis of naive and memory CD8 subsets.

As expected, more OA were cytomegalovirus (CMV) positive compared to YA (16/20 and 6/19, respectively, Additional Table S1). Taken together, our data confirm previously reported age-induced differences in immune composition and CMV serostatus. Furthermore, no differences in ratios of memory over naive CD4+ T cells were detected between CMV+ and CMV- YA and OA (Additional Fig. S2B).

### Effects of age on immune checkpoint expression in circulating CD4+ and CD8+ T cells

**Fig. 2:**
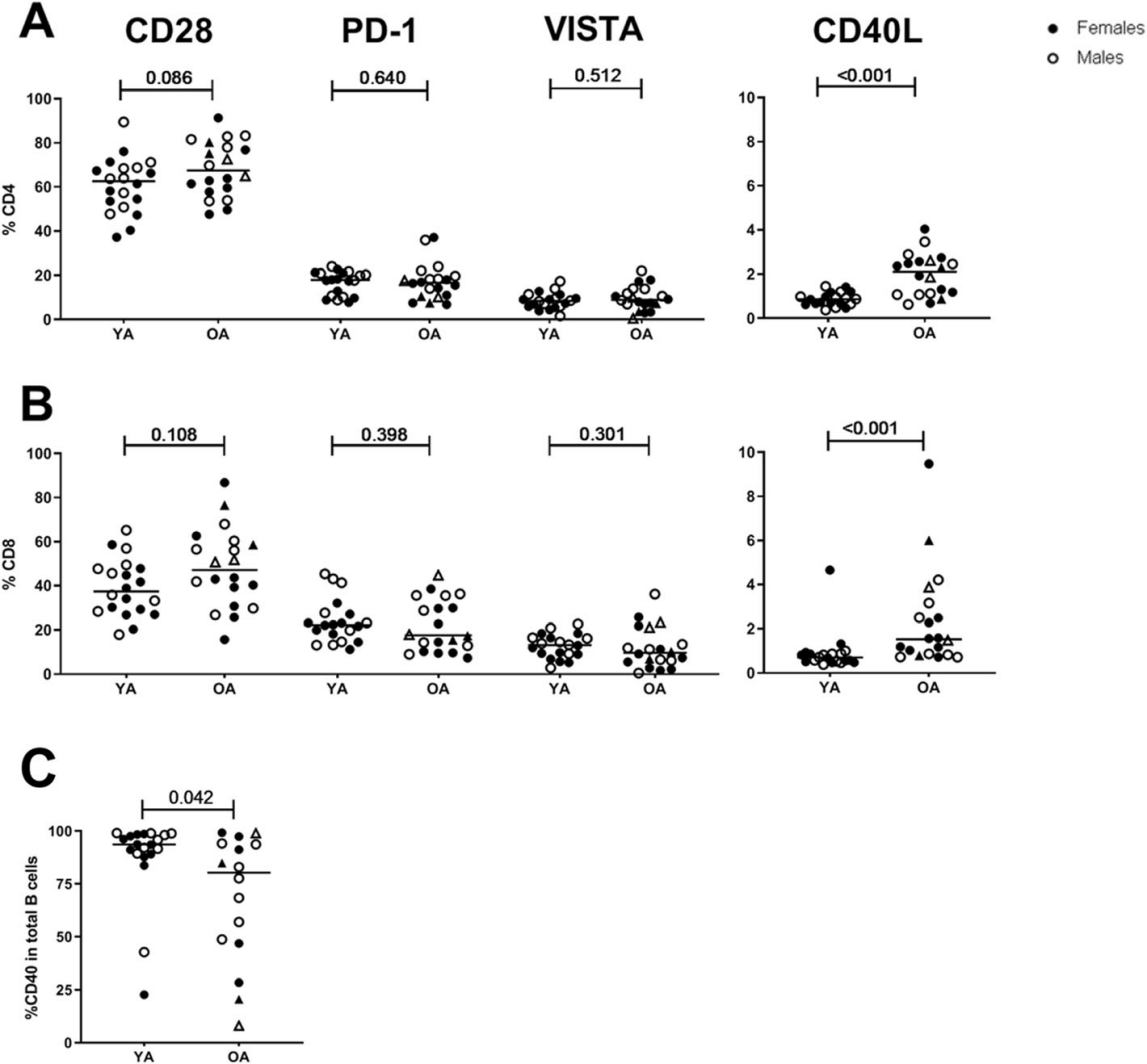
Immune checkpoint expression frequencies within CD4+ and CD8+ cells and B cells of young and older adults. Age effects on immune checkpoint expression were determined by flow cytometric staining of whole blood from young and older adults. Graphs represent percentages of CD28+, PD-1+, VISTA+ and CD40L+ cells within total CD4+ (**a**) and CD8+ T cells (**b**) and CD40+ cells within total B cells (**c**). Open and closed circles represent males and females, respectively. Open and closed triangles represent males (*n* = 2) and females (n = 2), respectively, that were 65 years of age or younger in the OA group. Horizontal bars reflect median percentages. The Mann-Whitney U test was used for comparison between young (YA, *n* = 20) and older (OA, *n* = 20) adults. P-values are indicated in the graphs

To study the effects of age on IC expression, frequencies of CD28+, PD-1+, VISTA+ and CD40L+ T cells were first determined within total circulating CD4+ and CD8+ cells of YA (*n* = 20) and OA (n = 20). Age did not have a strong effect on frequencies of CD28+, PD-1+ and VISTA+ cells within both CD4+ and CD8+ T-cell populations (Fig. 2a-b). However, age clearly affected CD40L expression, as an increase in the frequency of CD40L+ cells within both CD4+ and CD8+ cells was observed in OA (*p* < 0.001).

### Effects of age on CD40 expression by circulating B cells

CD40/CD40L interactions are important in T-and B-cell crosstalk. As we observed an increase in frequencies of CD40L + CD4+ cells in OA, we wondered how age would affect CD40 expression by B cells. Interestingly, we found a decrease in frequencies of CD40+ B cells in OA (*p* = 0.042, *n* = 16) (Fig. 2c**)**. Thus, age had opposite effects on CD40L and CD40 expression by T cells and B cells, respectively.

### Effects of age on immune checkpoint kinetics in circulating CD4+ and CD8+ T cells

**Fig. 3:**
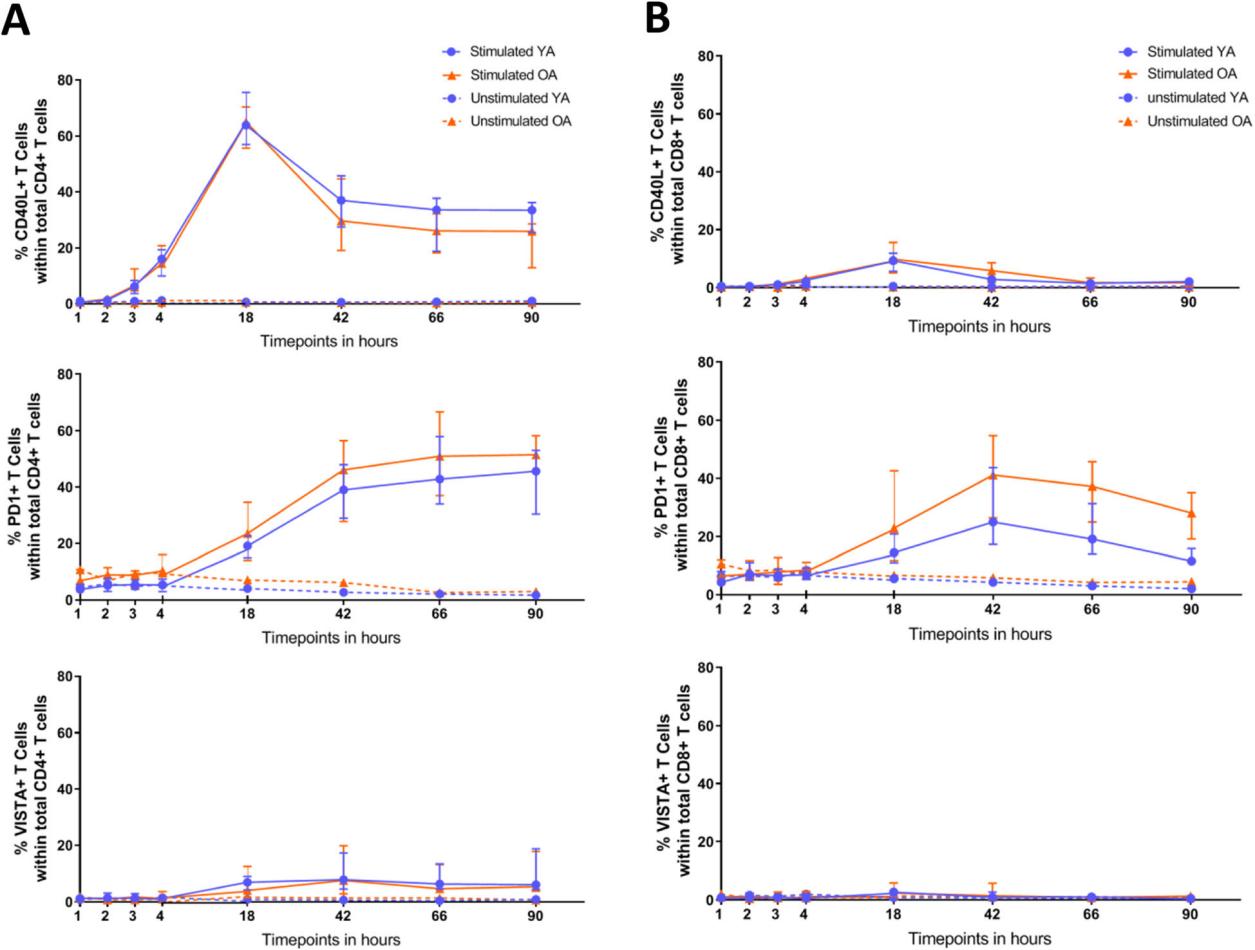
Kinetics of immune checkpoint expression. T cells were stimulated and immune checkpoint expression was measured at several time points thereafter. Graphs illustrate median percentages of CD40L, PD-1 and VISTA in total CD4+ cells (**a**) and CD8+ cells (**b**) at indicated time points (*n* = 10 young and 10 older adults). Blue and orange solid lines represent the median expression percentages of young (YA) and older adults (OA), respectively. Dotted blue and orange lines represent unstimulated cells. Error bars indicate interquartile range

To investigate whether the capacity to express CD40L, PD-1 and VISTA by CD4+ and CD8+ T cells upon stimulation is affected by age, we stimulated enriched CD4+ and CD8+ T-cell populations with anti-CD3 and anti-CD28 stimulation beads *ex vivo* and assessed frequencies of IC positive cells at 1, 2, 3, 4, 18, 42, 66 and 90 h thereafter. Figure 3a illustrates the kinetics of checkpoint expression by CD4+ cells of YA and OA. First, CD40L was most promptly upregulated and peaked at 18 h after stimulation with more than 60% of CD40L + CD4+ T cells. Hereafter, frequencies gradually declined with approximately 30–40% of CD4+ T cells remaining positive for CD40L at 90 h after stimulation. The kinetics of PD-1+ frequencies showed a somewhat slower increase and reached a plateau at around 40% of PD1 + CD4+ cells. The frequency of VISTA+ cells did not follow a clear pattern of upregulation after stimulation and remained low (< 10%) compared to the other ICs. No effects of age on IC expression kinetics by stimulated CD4+ cells were observed. In addition, we did not detect differences between males and females on the kinetics of IC expression (Additional Fig. S3). This would suggest that the capacity of T cells to upregulate immune checkpoints after antigenic stimulation is stable over age and comparable between males and females.

The pattern of IC upregulation by CD8+ cells was less pronounced than seen with the CD4+ subset (Fig. 3b). Also, whereas the frequency of PD-1+ cells within CD4+ cells stabilized after 42 h of stimulation, their frequencies within CD8+ T cells decreased somewhat after 42 h. Of note, CD8+ cells did not seem to upregulate VISTA at all.

### Effects of age on IC expression by CD4+ T-cell differentiation subsets

**Fig. 4:**
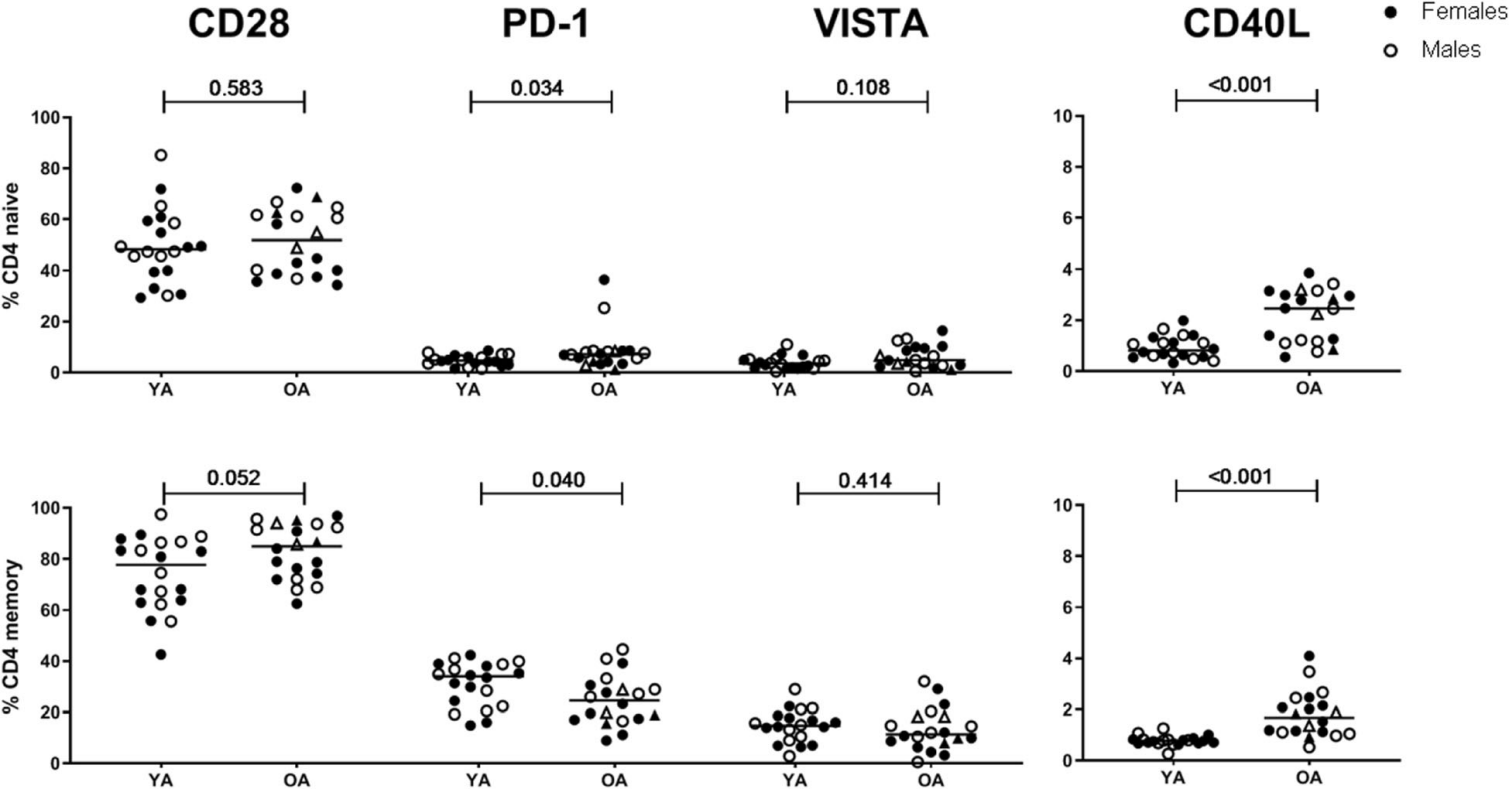
Immune checkpoint expression frequencies among naive and memory CD4+ cells of young and older adults. Age effects on CD28, PD-1, VISTA and CD40L expression within naive and memory CD4+ T cells were determined by flow cytometric staining of whole blood. Graphs represent percentages of immune checkpoints within total naive (CD45RA+) and memory (CD45RA-) CD4+ cells. Open and closed circles respectively represent males and females. Open and closed triangles represent males (*n* = 2) and females (n = 2), respectively, that were 65 years of age or younger in the OA group. Horizontal bars reflect median percentages. The Mann-Whitney U test was used for comparison between young (YA, *n* = 20) and older (OA, n = 20) adults. *P*-values are indicated in the graphs

Our data confirmed elevated memory/naive T-cell ratios in OA. Given these compositional changes, we analysed the expression of ICs on naive (CD45RA+) and memory (CD45RA-) CD4+ T cells. Here we found elevated frequencies of CD40L + CD4+ in both total naive and memory T cells (*p* < 0.001) in OA. Interestingly, subsetting of CD4 in naive and memory subsets, revealed that frequencies of PD-1+ cells were higher in total naive CD4+ (*p* = 0.034) in OA, whereas frequencies of PD-1+ cells were lower in total memory CD4+ cells (*p* = 0.04) in OA. In contrast, age did not seem to affect frequencies of CD28+ and VISTA+ cells among total naive and memory cells (Fig. 4). Age-associated effects on PD-1 and CD40L expression by naive and memory CD4+ T cells were not affected by CMV serostatus (Additional Fig. S4). No statistical differences in PD-1 expression were found after stratifying by CMV status, which is likely a result of a loss of power due to the small number of adults in both the CMV+ YA (*n* = 6) and CMV- OA (*n* = 4) groups. However, a similar pattern of expression was observed in CMV+ and CMV- YA and OA.

Next, we further subdivided naive and memory subsets based on CD25 expression using the Miyara classification to gain additional insight in IC expression by conventional memory and naive (fractions 4–7) versus regulatory CD4+ T subsets (fractions 1–3, Additional Fig. S5). In line with previous studies, our further subtyping of naive and memory fractions showed a lower frequency of cells in fraction 6 (*p* < 0.001, naive CD25- T cells) and a higher frequency of cells in the ageing-associated fraction 7 (p < 0.001, naive CD25^dim^ T cells) in OA [21, 22] (Additional Fig. S6). Interestingly, the higher frequencies of CD40L + CD4+ cells of OA (Fig. 2) were characteristic of all CD4+ fractions (Additional Fig. S7).

**Fig. 5:**
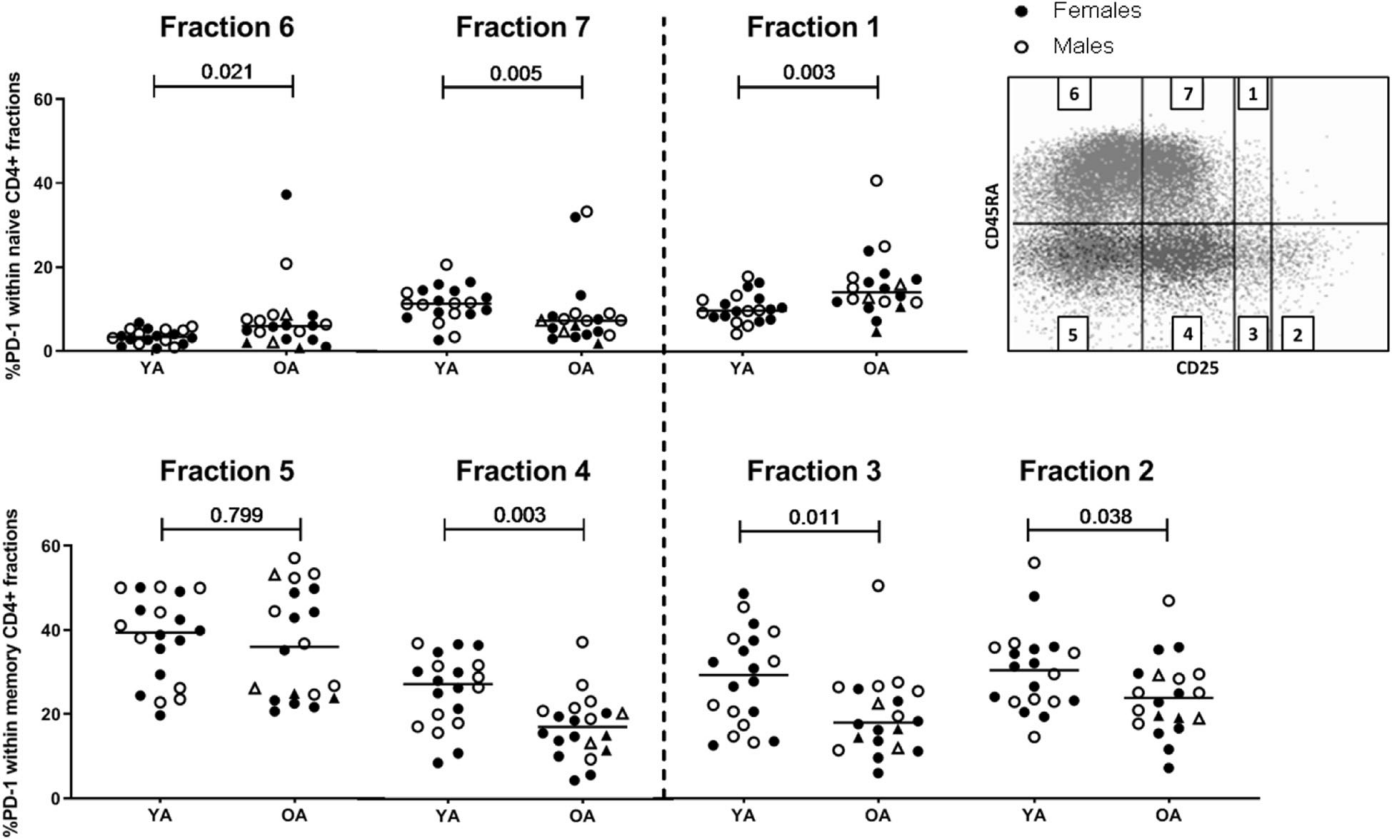
PD-1 expression frequencies among CD4+ differentiation subsets of young and older adults. Age effects on PD-1 expression were determined by flow cytometric staining of whole blood according to the Miyara classification employing CD45RA and CD25 [21, 22]. By staining for CD45RA and CD25 expression, the following seven fractions were distinguished: Memory CD25- (fraction 5), Memory CD25^dim^ (fraction 4), Memory CD25^int^ Treg (fraction 3), Memory CD25^high^ Treg (fraction 2), Naive CD25- (fraction 6), CD25^dim^ (fraction 7) and Naive CD25^int^ Treg (fraction 1). Graphs represent percentages of PD-1+ cells within the seven different naive and memory conventional and regulatory CD4+ fractions. Dashed line divides conventional memory and naive fractions from regulatory fractions. Open and closed circles respectively represent males and females. Horizontal bars reflect median percentages. Open and closed triangles represent males (n = 2) and females (n = 2), respectively, that were 65 years of age or younger in the OA group. The Mann-Whitney U test was used for comparison between young (YA, n = 20) and older (OA, n = 20) adults. P-values are indicated in the graphs

As we found that PD-1 frequencies were increased in naive CD4+ T cells but decreased in memory CD4+ T cells, further subsetting revealed that the increase in naive PD-1 + CD4+ T cells was mainly due to increased frequencies of PD-1+ cells in the naive/resting Treg subset (fraction 1, *p* = 0.003, CD25^int^ Treg). In addition, we found increased PD-1+ frequencies in the conventional naive CD4+ subset (fraction 6, *p* = 0.021, CD25-), whereas PD-1 frequencies were decreased in the ageing-associated naive CD25^dim^ subset (fraction 7, *p* = 0.005) in OA (Fig. 5, upper panel).

The decrease of PD-1 frequencies in memory CD4+ T cells was characteristic of all CD25+ memory subsets (fraction 4, (p = 0.003, CD25^dim^) and fraction 3 (*p* = 0.011, non-suppressive Tregs, CD25 ^int^), including Tregs fraction 2 (*p* = 0.038, activated Tregs, CD25^high^ Tregs)), whereas no differences in PD-1 frequencies were found in fraction 5 (*p* = 0.799, CD25-) (Fig. 5, lower panel).

Collectively, a more detailed analysis of CD4+ T-cell differentiation subsets revealed age- associated modulation of CD40L and PD-1 expression frequencies among both conventional and regulatory naive and memory subsets.

### Effects of sex on immune checkpoint expression by circulating CD4+ and CD8+ T cells

**Fig. 6:**
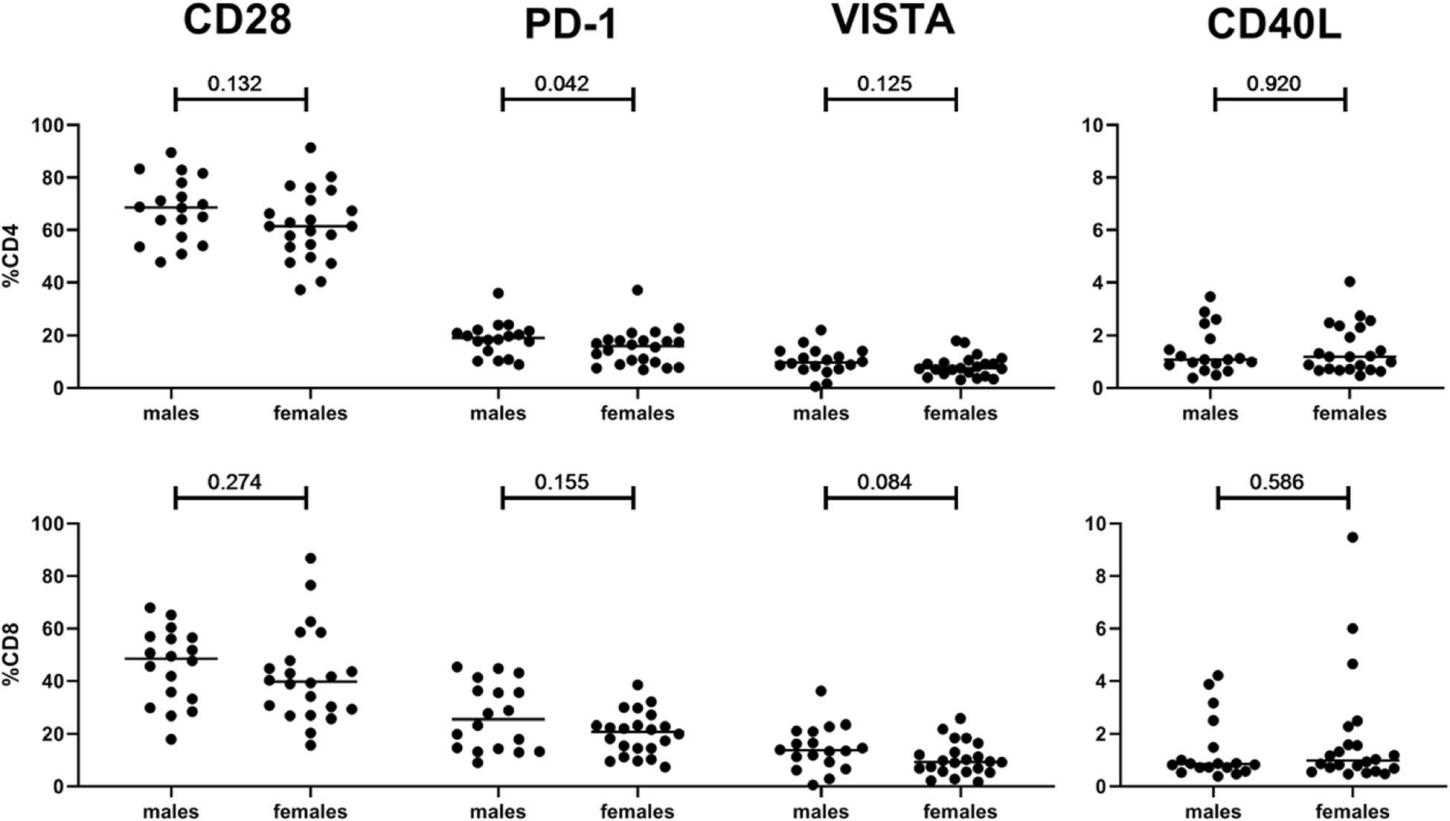
Effect of sex on CD28, PD-1, VISTA and CD40L expression. Sex effects on IC expression were determined by flow cytometric staining of whole blood. Graphs show the frequencies of ICs in males and females within total CD4+ fractions (**a**), and total CD8+ T cells (**b**). Horizontal bars reflect median percentages. The Mann-Whitney U test was used for comparison between two groups. P-values are indicated in the graphs

Sex effects on IC expression were determined by comparing IC expression by CD4+ and CD8+ cells between males and females. Sex did not affect CD28, VISTA and CD40L expression frequencies within total CD4+ and CD8+ cells nor PD-1+ frequencies within CD8+ cells. The frequencies of PD-1 expressing CD4+ T cells, however, were found to be decreased in females (*p* = 0.042) (Fig. 6). Next, when we compared effects of sex within each age group, lower frequencies of PD-1+ cells were detected in older females compared to older males (*p* = 0.038). Further subsetting based on CD45RA expression revealed that older females had lower frequencies of PD-1 + CD4+ memory cells (*p* = 0.046), whereas PD-1+ frequencies among CD4+ naive cells were comparable between older males and females (Additional Fig. S8). We also assessed interaction effects between age and sex regarding CD28, VISTA and CD40L expression but found no significant interaction effects in any CD4+ T cell subset and in total CD8+ T cells (Additional Table S3).

**Fig. 7:**
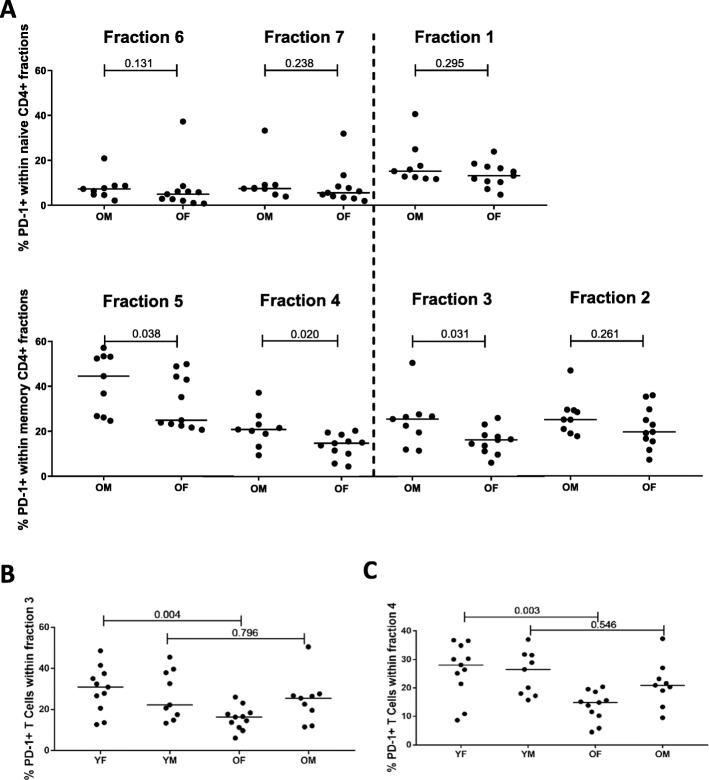
Effect of sex on PD-1 expression frequencies. Sex effects on PD-1 expression were determined by flow cytometric staining of whole blood. Graphs show the frequencies of PD-1+ cells in older males and females within different CD4+ fractions (**a**), and in young and older males and females in fraction 3 (**b**) and fraction 4 (**c**). Horizontal bars reflect median percentages. Dashed line divides conventional memory and naive fractions from regulatory fractions. For the comparison of 4 groups, a Kruskal-Wallis test was performed and found to be statistically significant in fraction 3 and 4 (*p* = 0.019 and *p* = 0.005). Each group consisted of *n* = 9 (males) and *n* = 11 (females) participants. The Mann-Whitney U test was used for comparison between two groups. P-values are indicated in the graphs

Next, we compared the frequencies of PD-1+ cells between older males and females following further CD4 subsetting (Fig. 7**)**. Indeed, no differences in PD-1+ frequencies among the naive fractions were noted. The lower frequencies of total PD-1 + CD4+ memory cells in older females, however, were due to lower frequencies in fraction 5 (*p* = 0.038), 4 (*p* = 0.020) and the non-suppressive Treg memory fraction 3 (*p* = 0.031). Fraction 2, comprising activated Tregs, was the only memory fraction in which PD-1 expression did not differ between the sexes. Therefore, the decrease of PD-1+ frequencies among cells in fraction 2 in OA (Fig. 5**)**, is solely an effect of age, rather than sex.

As our subsetting analysis had also revealed decreases of PD-1+ frequencies in memory CD4+ CD25^dim^ cells (fraction 4) and the non-suppressive Tregs (fraction 3) in OA compared to YA, we aimed to assess whether the age-associated decrease in these fractions is solely dependent on female sex. To this end, we assessed whether PD-1 expression was different between young and older females. Indeed, the age-associated decline in PD-1 frequencies in fractions 3 and 4 was found to associate with female, but not male, sex (fraction 3, *p* = 0.004 and fraction 4, *p* = 0.003, Fig. 7b-c).

Taken together, our data reveal an age- associated decline of PD-1 + CD4+ frequencies within defined memory subsets of older females.

## Discussion

In this study we show that both age and sex modulate expression of ICs by human T cells. More specifically, our study revealed an age-associated increase of CD40L + CD4+ T-cell frequencies and an age-associated decline of PD-1 + CD4+ memory T-cell frequencies in older females. The latter finding may aid the optimisation of PD-1 targeted immunotherapy and help the implementation of precision medicine in management of this vulnerable patient group.

By measuring IC expression within defined subsets of conventional and regulatory CD4+ T cells, we found that PD-1 expression on CD4+ memory cells is affected by both age and sex. More specifically, PD-1 expression frequencies among defined subsets of memory CD4+ cells were decreased upon age and were lower in older females than in older males. The observed effects of age on the non-suppressive Tregs (fraction 3) and the conventional memory CD25^dim^ CD4+ T cells (fraction 4) can be attributed solely to the decrease in PD-1 + CD4+ memory T-cell frequencies in older females. Previously, we reported on higher frequencies of PD-1-expressing CD4+ T cells in young, but not old patients with metastatic melanoma, data consistent with the current finding albeit that effects of sex were not documented [23].

PD-1 has been identified as a crucial IC in the regulation of the immune system and more specifically in preventing auto-immune diseases [24]. The rationale for this association arose from observations in PD-1 knockout mice, since these mice were prone to develop auto-immune diseases [25, 26]. Furthermore, blocking PD-1 in an experimental autoimmune encephalomyelitis (EAE) mouse model resulted in aggravated disease progression [27].

Since the older females in this study likely reached the post-menopausal status, the observed decrease in PD-1 + CD4+ frequencies in older females compared to young females, may be explained by a decrease in female sex hormones such as estrogens. Interestingly, previous studies in mice have suggested an effect of estrogen on PD-1 expression by Tregs. Firstly, estrogen (17-beta-estradiol) treatment increased intracellular PD-1 expression in Treg cells in mice, whereas oestrogen receptor knockout mice had reduced PD-1 expression and reduced Treg suppression [28]. Secondly, the PD-1 induction in the Treg compartment by estrogen correlated with better suppression and hence EAE protection whereas Treg deficient mice appeared to be unprotected against EAE. Lastly, estrogen reduced IL-17 levels in immunized mice, whereas estrogen did not reduce IL-17 levels in PD-1 deficient mice. These results suggest suppression of IL-17 by estrogen-induced PD-1 + Tregs [29].

Thus, the mouse studies show that PD-1 expression by Tregs can be modulated by estrogens with clear consequences for experimental autoimmunity. It remains to be elucidated if female sex hormones modulate PD-1 expression in humans and if this is limited to the Treg compartment. Additionally, other (hormonal) factors are likely involved in the regulation of PD-1 expression as well, as we did not observe lower PD-1 frequencies among CD4+ T cells from older males. Nevertheless, some auto-inflammatory diseases such as giant cell arteritis, a large vessel vasculitis typically affecting older females, and late-onset rheumatoid arthritis could be related to a post-menopausal decrease in PD-1 expression. Clearly, this will require further dedicated studies.

Importantly, age- and sex-associated differences in PD-1 expression frequencies could have implications for PD-1 blockade therapies in cancer. These therapies typically reverse the state of exhaustion of tumour specific effector T cells. Several papers have reviewed the efficacy of PD-1 blocking therapies in clinical trials having included older adults and found contradictory and inconsistent results. For some cancers such as head and neck, non-small cell lung cancer and metastatic renal cell carcinoma, the efficacy of IC-blocking therapies in older adults seemed to be decreased [30], but often no age-associated differences were found [31, 32]. In addition, some studies noted increased efficacies with ageing [33]. Overall, it should be appreciated that older patients (> 65 years) have been under-represented in many clinical trials, and that those who were included may not be representative of the older population in general [30]. In addition, a meta-analysis addressing sex-dependent effects of IC-blocking therapy revealed a lower efficacy in women than in men. Here, differences between young and older patients were not addressed [34]. Together, these findings indicate that more studies are needed to determine the efficacy and toxicity of IC-blocking therapy in young and older males and females.

Frequencies of CD40L+ T cells were impacted by age as well. Although differences in frequencies of CD40L+ cells between young and older adults appeared to be subtle, the increase in the older group was consistent and seen in every subset of CD4+ T cells. As CD40L pairs with CD40 in T-cell/B-cell interactions, an increased expression of CD40L could lead to an increased B-cell response [35, 36]. This, however, is contradictory with the notion that the humoral response wanes with ageing. Indeed, the observed increase in CD40L+ T-cell frequencies was paralleled by a decrease in CD40+ B-cell frequencies in older adults, suggesting that upregulation of CD40L may be a compensatory mechanism. CD40L/CD40 interactions have been found to be important in autoimmune diseases such as SLE. In SLE, CD40L expression is increased on circulating B- and T cells but data on CD40 expression levels have not been reported [37]. Elevated CD40L levels have been suggested to cause an amplification of the immune response and an increase in autoantibodies [37–39]. Whether the observed increase of CD40L + CD4+ T cells in older adults is solely a compensatory mechanism or has consequences for immune function and humoral responses remains to be further investigated.

The kinetics of IC-expression appeared to be different between CD4+ and CD8+ cells. In line with our observations, an in vitro study performed by Sabins et al. showed higher PD-1 expression by CD4+ cells than by CD8+ cells [40]. Although differences were seen in IC-expression by circulating T cells, the capacity of T cells to upregulate ICs upon stimulation *ex vivo* appeared to be unaffected by age and sex.

This study has some limitations. First, we report results of a relatively small number of healthy donors. Results in this study should therefore be interpreted with caution and be seen as a first step towards more information on age and sex effects on IC expression and kinetics. Secondly, while assessing the kinetics of IC-expression, we did not determine other characteristics such as proliferative capacity and cytokine production, which could have revealed more clues as to how age and sex affect T-cell function. Furthermore, it remains to be established if the decrease in PD-1-expressing CD4+ memory T cell frequencies as seen in older females translates to T cell functional changes such as a potential increase in proliferative capacity. Thirdly, our study was not designed to conclude on the effects of CMV serostatus on T-cell IC expression, but we were able to exclude CMV carriage as a confounder in our study, as similar patterns were observed in young and older CMV+ and CMV- donors. Furthermore, we did not observe a decline in CD28 frequencies upon age which was expected for at least the CD8 population [20]. One explanation could be that four of our OA volunteers were relatively young (between 54 and 65) and had relatively high CD28+ frequencies among CD4+ and CD8+ T cells which may have skewed the data. Lastly, our methods did not allow for accurate analysis of the average per cell IC-expression (MFI), which could give additional information when analysing the effects of combined positive and negative IC expression.

## Conclusions

In this study, we provide evidence that both age and sex modulate expression of ICs by human T cells. Our study revealed an age-associated increase of CD40L + CD4+ T-cell frequencies and an age-associated decrease of CD40+ B-cell frequencies, suggesting modulation of the CD40L-CD40 interaction with age. Importantly, we documented an age-associated decline of PD-1 + CD4+ memory T cells in older females.

We hope that our findings will prompt further research as more knowledge may aid the optimisation of vaccination and targeted immunotherapy and move the field towards precision medicine in the management of older patient groups.

## Materials and methods

### Study population

To study the effect of age and sex on immune checkpoint expression, 20 healthy young adults (YA, median age and range in years: 26 (20–31), male/ female ratio 9/11) and 20 healthy older adults (OA, median age and range in years: 72 (54–86), male/female ratio 9/11) were enrolled in this study (Additional Table S1). Since CMV serostatus has known effects on immune function and as CMV seropositivity increases with age [41], CMV serostatus was determined in all participants as described before [42]. CD40 expression by B cells was measured in all 20 YA and in 16 of the 20 OA. The kinetics of immune checkpoint expression was determined in a subset of donors consisting of 10 healthy young and 10 healthy older adults (median age and range in years YA: 27 (20–31) and OA: 72 (63–83)), each group consisting of 5 males and 5 females (Additional Table S1).

The participants’ health status was confirmed by a clinician through questionnaires in young donors and by physical examination, lab tests and questionnaires in older adults. Donors need to fulfil the adapted SENIEUR criteria for health status [43]. Volunteers who were diagnosed with inflammatory or infectious conditions or treated with immunomodulatory drugs were not included. The constitutional review board of the UMCG approved this study (METc2012/375) and all donors gave their written informed consent prior to blood withdrawal.

### Quantification of leukocytes

Absolute leukocyte counts of the majority of donors (YA: *n* = 14, median age and range in years: 26 (30–31) OA: n = 14, median age and range in years: 74 (57–86)) were measured in EDTA blood according to the MultiTest TruCount method (BD Biosciences, Durham, NC, USA) according to the manufacturer’s instructions. When absolute counts of an individual donor were measured on multiple days or when the individual donor’s age differed among the different experiments, the absolute counts and ages were averaged (Additional Table S1).

### Immune checkpoint expression by whole blood immune cells

To measure IC expression on circulating immune cells, fresh blood collected in EDTA tubes was washed twice with PBS and stained with monoclonal antibodies detecting CD3, CD4, CD45RA, CD25, CD19, CD28, PD-1, VISTA, CD40 and CD40L for 15 min (Additional Table S2). After surface staining, cells were fixed and red blood cells were lysed with FACS lysing solution (BD Biosciences, 1:10 dilution). Samples were measured on a BD LSR-II flow cytometer. Setting of gates was determined by isotype controls and IC expression was presented as the percentage of positive cells within CD4+ and CD8+ T cells and CD19+ B cells. The use of isotype controls for setting gates was assessed and confirmed by fluorescence minus one controls. CD4+ cells were further subclassified into seven fractions by virtue of CD45RA and CD25 expression, based on the classification criteria reported by Miyara et al. (2009) and adapted by van der Geest et al. (2014) (Additional Fig. S5) [21, 22]. This method allowed the classification of naive and memory CD4+ T cells as CD45RA+ and CD45RA-, respectively. In addition, CD25 allowed for subsetting of resting and activated conventional and regulatory naive and memory subsets. Fraction 1, 2 and 3 identify resting/naive Tregs, activated/memory Tregs and non-suppressive or cytokine secreting Tregs, respectively. Fraction 4 and 5 comprise conventional memory cells with dim CD25 and memory cells lacking CD25 expression, respectively. Fraction 6 and 7 comprise conventional naive cells and age-associated naive CD25^dim^ T cells, respectively.

### Kinetics of immune checkpoint expression *ex vivo*

Expression kinetics were determined by measuring ICs at defined time points after T-cell stimulation *ex vivo*. Briefly, peripheral blood mononuclear cells were isolated from blood collected in heparin tubes by density gradient centrifugation using Lymphoprep (Alere Technologies AS, Oslo, Norway). Next, T cells were isolated by negative selection using the MagniSort Human T cell Enrichment Kit (Thermo Fisher Scientific, Waltham, MA., USA) or the EasySep™ Human T cell isolation kit (Stemcell Technologies, Vancouver, Canada) (results were found to be similar for both kits, purity ≥92%) according to manufacturer’s instructions. After T-cell enrichment, 0.5 × 10^6^ T cells were added to 1 mL of Roswell Park Memorial Institute (RPMI) culture medium with HEPES and L-Glutamine (Lonza, Basel, Switzerland) supplemented with gentamycin (Lonza), in round-bottomed polypropylene tubes. Gibco Dynabeads™ Human T-activator anti-CD3/anti-CD28 (ThermoFisher, Waltham USA) were added to T cells in a ratio of 1:5. Stimulated T cells were then incubated at 37 °C, 5% CO2 and collected after 1,2,3,4, 18, 42, 66 and 90 h and stained for CD8, CD45RA, CD25, PD-1, VISTA, and CD40L. In parallel, unstimulated T cells were also assessed for the same ICs including CD28 at each time point (See additional Table 2 for the antibodies used). Staining was performed as described above.

### Data analysis and statistics

Flow cytometry data was analysed with Kaluza Analysis Software (Beckman Coulter, California, USA) and graphs were created with GraphPad Prism 7 (GraphPad Software, San Diego, USA). Two-tailed Kruskal-Wallis tests were performed when multiple groups were compared and Mann-Whitney U tests for comparing two groups. Interaction effects of age and sex were explored via factorial ANOVA. All results were considered statistically significant when *p* < 0.05.

## Supplementary Information

**Additional file 1:** **Table S1** Overview of the Study population and samples used. **Table S2** Fluorescent-conjugated monoclonal antibodies. **Table S3** Results of factorial ANOVA to determine interaction effects of age and sex. **Fig. S1** Absolute cell counts in peripheral blood of females and males. **Fig. S2** Ratios of memory/naive CD4+ T cells in young and older adults and effect of CMV serostatus. **Fig. S3** Kinetics of immune checkpoint expression in females and males. **Fig. S4** PD-1 and CD40L expression by naive and memory CD4+ T cells in young and older CMV+ and CMV- adults. **Fig. S5** Flow cytometric strategy for determining IC expression by CD4+ and CD8+ T cells and B cells. **Fig. S6** Proportions of memory and naive fractions within CD4+ T cells in young and older adults. **Fig. S7** Percentages of CD40L+ cells within fractions of CD4+ T cells in young and older adults. **Fig. S8** Percentages of PD-1+ cells within total, naive and memory CD4+ T cells in older males and females.

## Data Availability

The datasets used and/or analysed during the current study are available from the corresponding author on reasonable request.

## Abbreviations

YA: Young adults
OA: Older adults
CMV: Cytomegalovirus
ICs: Immune checkpoints
irAEs: Immune-related adverse events
PD-1: Programmed Death 1
SLE: Systemic lupus erythematosus
T_EM_: Effector memory T cell
Tregs: Regulatory T cells
VISTA: V-domain Ig suppressor of T-cell activation
