# Supplementary material for: Effect of age and sex on immune checkpoint expression and kinetics in human T cells
Source: Immun Ageing. 2020 Nov 4;17:32. doi: 10.1186/s12979-020-00203-y (PMC7640492; doi:10.1186/s12979-020-00203-y)
Supplement: Supplementary file 1 — Additional file 1: Table S1 Overview of the Study population and samples used. Table S2 Fluorescent-conjugated monoclonal antibodies. Table S3 Results of factorial ANOVA to determine interaction effects of age and sex. Fig. S1 Absolute cell counts in peripheral blood of females and males. Fig. S2 Ratios of memory/naive CD4+ T cells in young and older adults and effect of CMV serostatus. Fig. S3 Kinetics of immune checkpoint expression in females and males. Fig. S4 PD-1 and CD40L expression by naive and memory CD4+ T cells in young and older CMV+ and CMV- adults. Fig. S5 Flow cytometric strategy for determining IC expression by CD4+ and CD8+ T cells and B cells. Fig. S6 Proportions of memory and naive fractions within CD4+ T cells in young and older adults. Fig. S7 Percentages of CD40L+ cells within fractions of CD4+ T cells in young and older adults. Fig. S8 Percentages of PD-1+ cells within total, naive and memory CD4+ T cells in older males and females. [file 12979_2020_203_MOESM1_ESM.docx]

**Additional information**

**Additional Table S1: Overview of the Study population and samples used.**

| Donor code | Age  (years) | M/F | CMV* | BMI | Ethnicity | TFI** | Absolute  counts | Whole blood  staining | | IC kinetics |
| --- | --- | --- | --- | --- | --- | --- | --- | --- | --- | --- |
| Young Adults: |  |  |  |  |  |  |  |  |  | |
| HC1 | 27 | M | Neg | 22.1 | Caucasian |  |  | X | X | |
| HC2 | 28 | M | Pos | 24.8 | Caucasian |  |  | X | X | |
| HC3 | 31 | F | Pos | 21.5 | Other |  | X | X | X | |
| HC4 | 23 | F | Neg | 21.8 | Caucasian |  |  | X | X | |
| HC5 | 26 | M | Neg | 22.6 | Caucasian |  |  | X |  | |
| HC6 | 20 | F | Neg | 23.9 | Caucasian |  |  | X | X | |
| HC7 | 26 | F | Pos | 20.3 | Caucasian |  |  | X |  | |
| HC8 | 28 | M | Neg | 21.4 | Caucasian |  | X | X | X | |
| HC9 | 24 | F | Neg | 22.1 | Caucasian |  | X | X |  | |
| HC10 | 29 | F | Neg | 21.0 | Caucasian |  | X | X | X | |
| HC11 | 23 | F | Neg | 30.1 | Caucasian |  | X | X | X | |
| HC12 | 22 | M | Neg | 20.9 | Caucasian |  | X | X | X | |
| HC13 | 26 | M | Neg | 20.5 | Caucasian |  | X | X | X | |
| HC14 | 29 | M | pos | 22.3 | Asian |  | X | X |  | |
| HC15 | 21 | F | neg | 19.6 | Caucasian |  | X | X |  | |
| HC16 | 26 | F | pos | 24.6 | Caucasian |  | X | X |  | |
| HC17 | 28 | M | pos | 29.4 | Other |  | X | X |  | |
| HC18 | 26 | M | neg | 18.6 | Caucasian |  | X | X |  | |
| HC19 | 21 | F | inc | 19.2 | Caucasian |  | X | X |  | |
| HC20 | 28 | F | neg | 28.7 | Caucasian |  | X | X |  | |
|  |  |  |  |  |  |  |  |  |  | |
| Older Adults: |  |  |  |  |  |  |  |  |  | |
| OHC1 | 85 | M | Pos | 22.7 | Caucasian | 5 | X | X |  | |
| OHC2 | 86 | F | Neg | 47.7 | Caucasian | 12 | X | X |  | |
| OHC3 | 71 | M | Pos | 24.3 | Caucasian | 1 | X | X |  | |
| OHC4 | 77 | M | Pos | 28.4 | Caucasian | 10 | X | X |  | |
| OHC5 | 68 | F | Pos | 30.1 | Caucasian | 3 | X | X | X | |
| OHC6 | 72 | M | Pos | 24.5*** | Caucasian | 1*** | X | X | X | |
| OHC7 | 54 | F | Pos | 19.6 | Caucasian |  |  | X |  | |
| OHC8 | 71 | F | Pos | 21.8 | Caucasian | 1 |  | X |  | |
| OHC9 | 77 | F | Pos | 25.9 | Caucasian | 2 |  | X |  | |
| OHC10 | 78 | F | Pos | 23.1 | Caucasian | 3 |  | X |  | |
| OHC11 | 72 | F | Neg | 36.3 | Caucasian | 4 |  | X |  | |
| OHC12 | 65 | M | Neg | 24.7 | Caucasian |  |  | X | X | |
| OHC13 | 63 | F | Pos | 24.8 | Caucasian |  | X | X | X | |
| OHC14 | 81 | M | Pos |  | Caucasian |  | X | X | X | |
| OHC15 | 82 | F | Pos |  | Caucasian |  | X | X | X | |
| OHC16 | 76 | F | Pos | 21.3 | Caucasian | 3 | X | X | X | |
| OHC17 | 83 | M | Pos | 24.8 | Caucasian | 8 | X | X | X | |
| OHC18 | 57 | M | Neg | 24.7 | Caucasian |  | X | X |  | |
| OHC19 | 66 | F | Pos | 26.7 | Caucasian | 3 | X | X | X | |
| OHC20 | 72 | M | pos | 27.1 | Caucasian | 1 | X | X | X | |

**M = male. F = female. Absolute counts were determined by TruCount. X indicates that the sample was used in the experiments indicated.**

*** CMV Pos or Neg indicates that the CMV serostatus is positive or negative. Inc= CMV status was inconclusive.**

**** Tilburg frailty indicator. Score range: 0-15. Adults with a score of ≥5 are considered frail.**

***** Assessed one year prior to blood collection**

**Additional Table S2. Fluorescent-conjugated monoclonal antibodies.**

| **Antibody** | **Colour** | **Clone** | **Company** | **Amount** | **Isotype control** |
| --- | --- | --- | --- | --- | --- |
| VISTA | PE | 730804 | R&D | 10 μL | PE Mouse IgG2B, κ |
| CD279 (PD-1) | BV605 | EH12.2H7 | BioLegend | 2 μL | BV605 Mouse IgG1, κ |
| CD152 (CTLA-4) | PE- Cyanine7 | 14D3 | eBioscience | 2 μL | PE-Cy7 Mouse IgG2a, κ |
| CD28 | APC-H7 | CD28.2 | BD Biosciences | 2 μL | APC-H7 Mouse IgG1, κ |
| CD154 (CD40L) | APC | 24-31 | eBioscience | 2 μL | APC Mouse IgG1, κ |
| CD25 | BV421 | BC96 | Biolegend | 2 μL | BV421 Mouse IgG1 , κ |
| CD45RA | BUV737 | HI100 | BD Biosciences | 2 μL | BUV737 Mouse IgG2b, κ |
| CD16 | BUV395 | 3G8 | BD Biosciences | 3 μL | BUV395 Mouse IgG1, κ |
| CD40 | APC-Cy7 | 5C3 | Biolegend | 2 μL | APC/Cy7 Mouse IgG1, κ |
| CD8 | APC-H7 | SK1 | BD Biosciences | 5 μL | X |
| CD19 | BV421 | HIB19 | BD Biosciences | 2 μL | X |
| CD3 | AF700 | UCHT1 | BD Biosciences | 5 μL | X |
| CD4 | PerCP Cy 5.5 | OKT-04 | BioLegend | 2 μL | X |
| CD56 | BV785 | 5.1H11 | Biolegend | 2 μL | X |

**Additional Table S3: Results of factorial ANOVA to determine interaction effects of age and sex***

|  | CD28 | VISTA | CD40L |
| --- | --- | --- | --- |
| total CD4 | 0.815 | 0.768 | 0.749 |
| naive CD4 | 0.937 | 0.535 | 0.669 |
| memory CD4 | 0.672 | 0.528 | 0.685 |
| fr1 CD4 | 0.748 | 0.881 | 0.637 |
| fr2 CD4 | 0.974 | 0.821 | 0.198 |
| fr3 CD4 | 0.797 | 0.332 | 0.647 |
| fr4 CD4 | 0.740 | 0.420 | 0.809 |
| fr5 CD4 | 0.543 | 0.569 | 0.895 |
| fr6 CD4 | 0.903 | 0.678 | 0.468 |
| fr7 CD4 | 0.645 | 0.470 | 0.862 |
| total CD8 | 0.669 | 0.704 | 0.894 |

*** interaction effects of age and sex were determined by factorial ANOVA, values represent p-values**

*
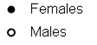
* ***Additional figure S1.*** ***Absolute cell counts in peripheral blood of females and males.*** *Absolute cell counts of total lymphocytes, CD3+, CD4+ and CD8+ T cells, NK cells and B cells were determined by TruCount. Horizontal bars reflect median values. Light pink areas represent values outside the reference range. Reference values were provided by the department of Laboratory Medicine (UMCG). The Mann-Whitney U test was used for comparisons between females (n=14) and males n=14). P-values are indicated in the graphs.*

**B**

**A**

**
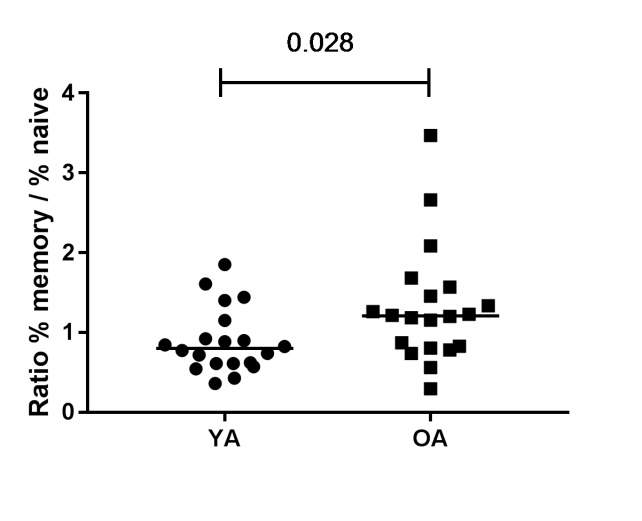
** **
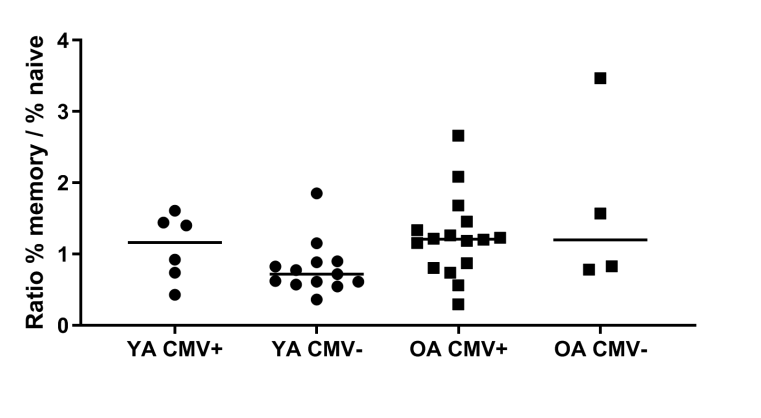
**

***Additional figure S2. Ratios of memory/naive CD4+ T cells in young and older adults*** ***and effect of CMV serostatus.*** *The frequencies of memory and naive cells in young and older adults (A) and young and older CMV+ and CMV- adults (B) were determined by flow cytometric staining of whole blood using CD45RA. Horizontal bars reflect median ratios. The Mann-Whitney U test was used to compare ratios of memory over naive cells between young (YA, n=20) and older (OA, n=20) adults. The P-value is indicated in the graph. The Kruskal- Wallis test detected no differences between the groups in the ratios of memory/naive CD4+ T cells in CMV+ YA (n=6), CMV- YA (n=13), CMV+OA (n=16) and CMV- OA (n=4).*

*
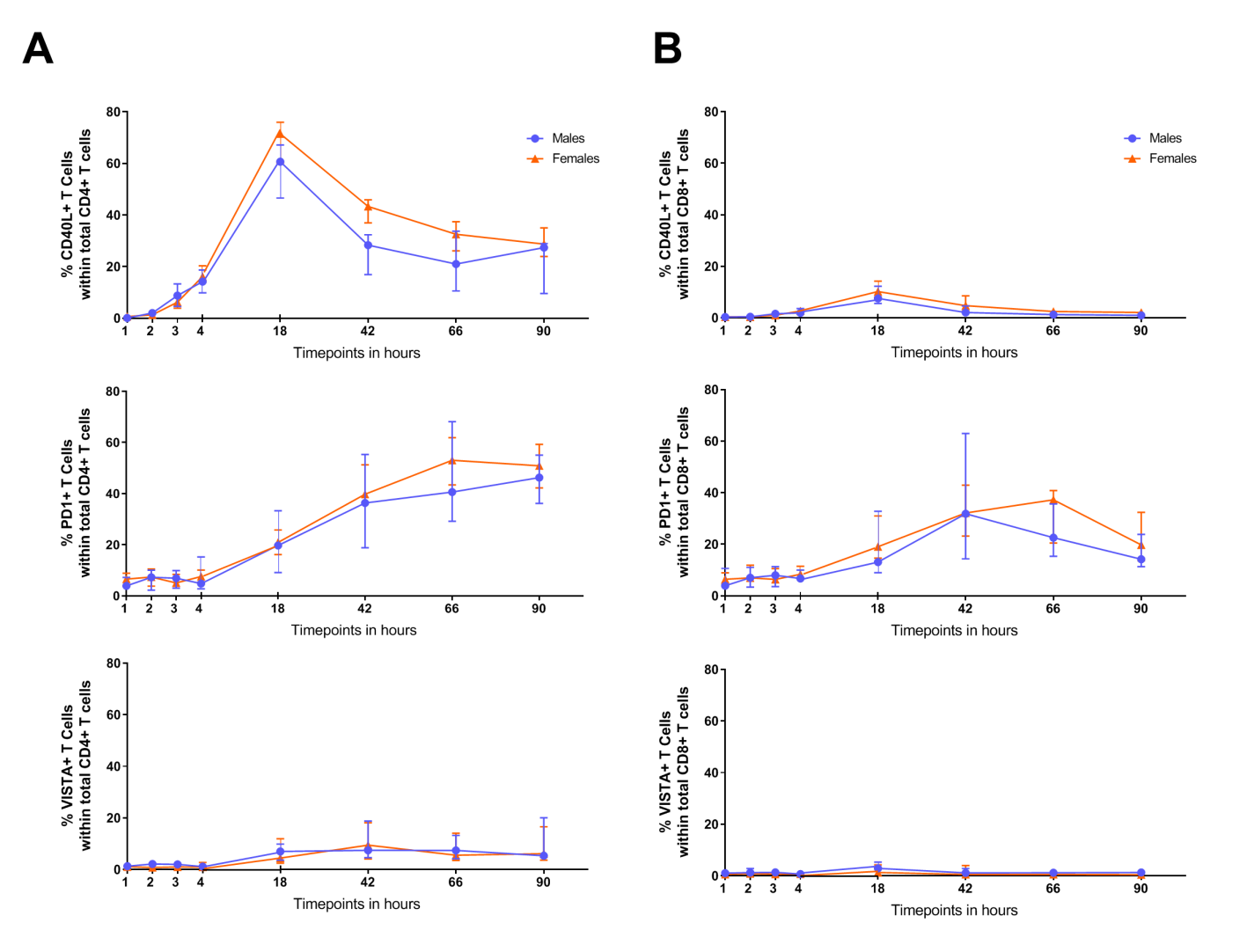
*

***Additional figure S3: Kinetics of immune checkpoint expression in females and males.*** *T cells were stimulated and immune checkpoint expression was measured at several time points thereafter.* *Graphs illustrate median percentages of CD40L, PD-1 and VISTA in total CD4+ cells (A) and CD8+ cells (B) at indicated time points (n=10 males and 10 females). Blue and orange lines represent the median expression percentages of males and females, respectively. Error bars indicate interquartile range.*

*
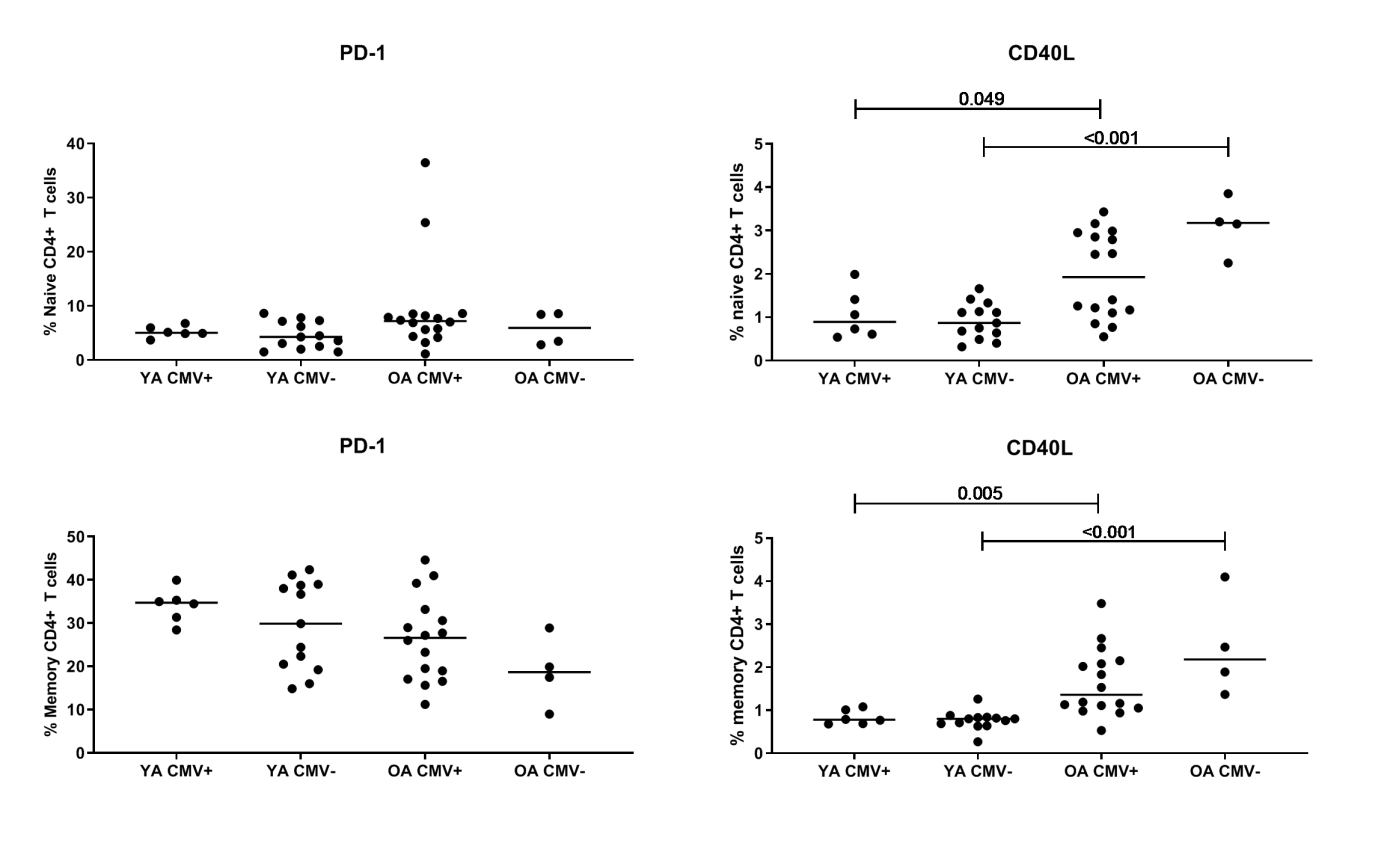
*

***Additional figure S4: PD-1 and CD40L expression by naive and memory CD4+ T cells in young and older CMV+ and CMV- adults.*** *The frequencies of PD-1 and CD40L in memory and naive cells in young and older CMV+ and CMV- adults were determined by flow cytometric staining of whole blood using CD45RA. Horizontal bars reflect median ratios. The Kruskal- Wallis test revealed differences between groups in CD40L expression but not in PD-1 expression in naive and memory CD4+ T cells in CMV+ YA (n=6), CMV- YA (n=13), CMV+OA (n=16) and CMV- OA (n=4). The Mann-Whitney U test was used to compare frequencies of CD40L+ cells between CMV+ YA and CMV+ OA and CMV- YA and CMV- OA. P-values are indicated in the graph.*

**
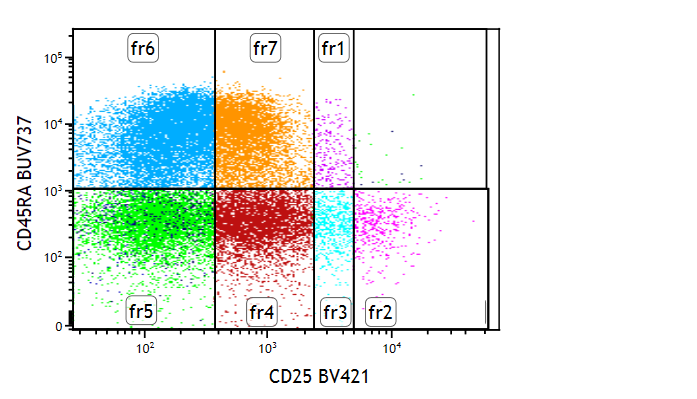
**
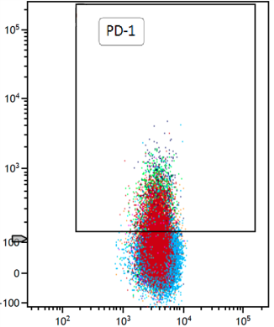

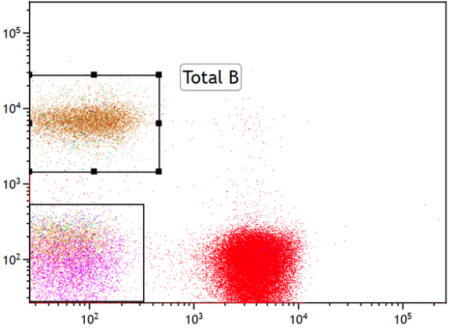

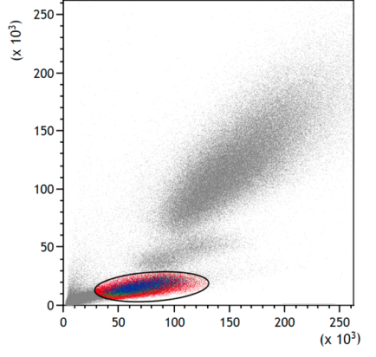


**E**

**D**

**B**

**C**

**A**

**SSC**

**CD3 AF700**

**FSC**

**CD19 BV421**


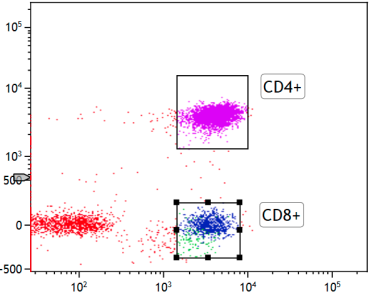


**CD4 PerCP-Cy5.5**

**CD45RA BUV737**

**PD-1 BV605**

**Memory**

**Naive**

**CD25 BV421**

**CD3 AF700**

***Additional figure S5. Flow cytometric strategy for determining IC expression by CD4+ and CD8+ T cells and B cells.*** *Lymphocytes were determined based on their forward and side scatter (A). Then, B cells were defined as CD19+ and CD3- (B). CD4+ T cells were defined as CD4+CD3+ and CD8+ as CD4-CD3+ (C). CD4+ T cells were further defined using CD45RA and CD25. By staining for CD45RA and CD25 expression* (21,22)*, seven fractions were distinguished (D). Memory fractions CD45RA-: Memory CD25- (fraction 5), Memory CD25^dim^ (fraction 4), Memory CD25^int^ Treg (fraction 3) and Memory CD25^high^ Treg (fraction 2). Naive fractions CD45RA+: Naive CD25- (fraction 6), CD25^dim^ (fraction 7) and Naive CD25^int^ Treg (fraction 1).* *IC expression is determined by gating appropriate isotype controls.* *The example shows PD-1 staining on CD4+ T cells (E).*

**
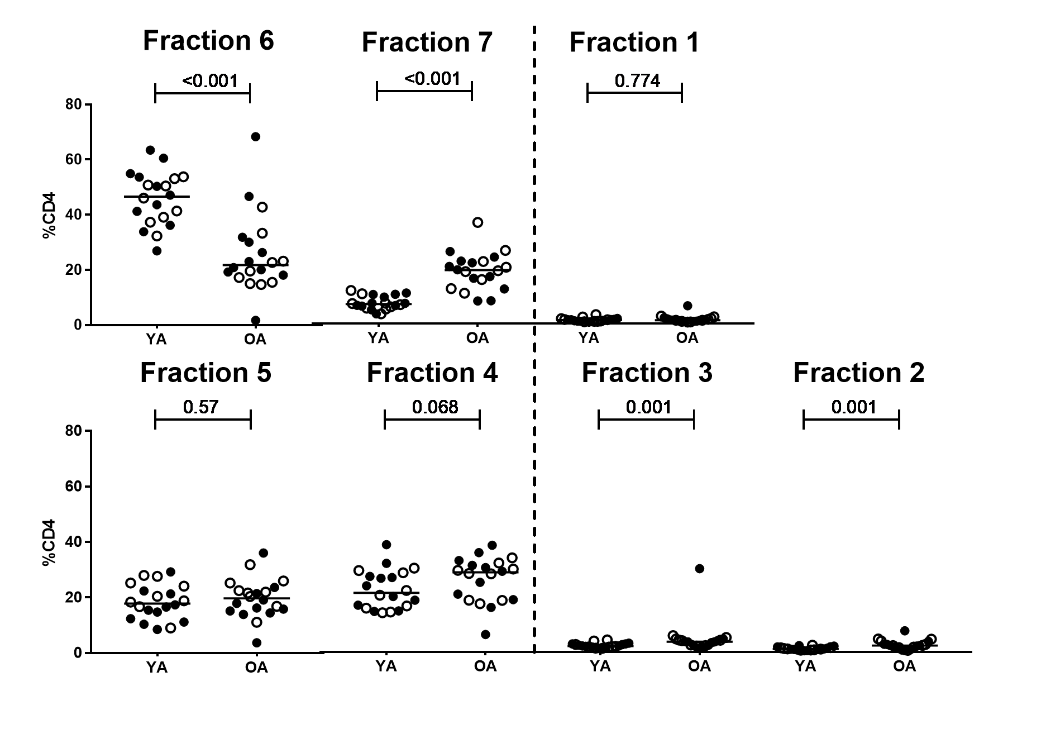
**

***Additional figure S6. Proportions of memory and naive fractions within CD4+ T cells in young and older adults.*** *The frequencies of memory and naive fractions were determined by flow cytometric staining of whole blood with CD25 and CD45RA. Horizontal bars reflect median percentages. Dashed line divides memory and naive fractions from regulatory fractions. The Mann-Whitney U test was used to compare frequencies between young (YA, n=20) and older (OA, n=20) adults. P-value is indicated in the graph.*

**
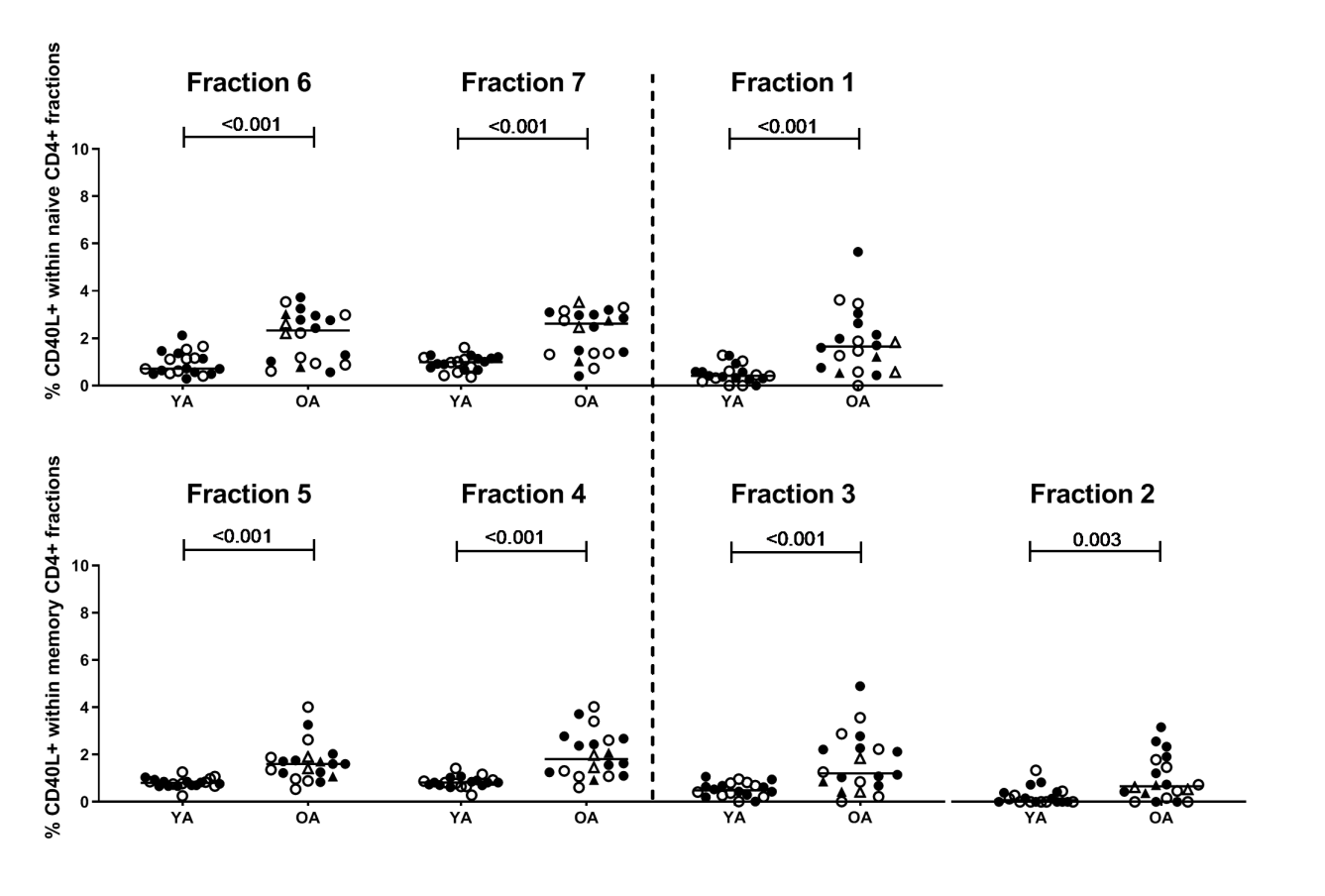
**

***Additional figure S7 Percentages of CD40L+ cells within fractions of CD4+ T cells in young and older adults.*** *Age effects on CD40L expression were determined by flow cytometric staining of whole blood. Open and closed circles respectively represent males and females. Open and closed triangles represent males (n=2) and females (n=2), respectively, that were 65 years of age or younger in the OA group. Horizontal bars reflect median percentages. Dashed line divides memory and naive fractions from regulatory fractions. The Mann-Whitney U test was used to compare frequencies of CD40L+ cells between young (YA, n=20) and older (OA, n=20) adults. P-values are indicated in the graphs.*


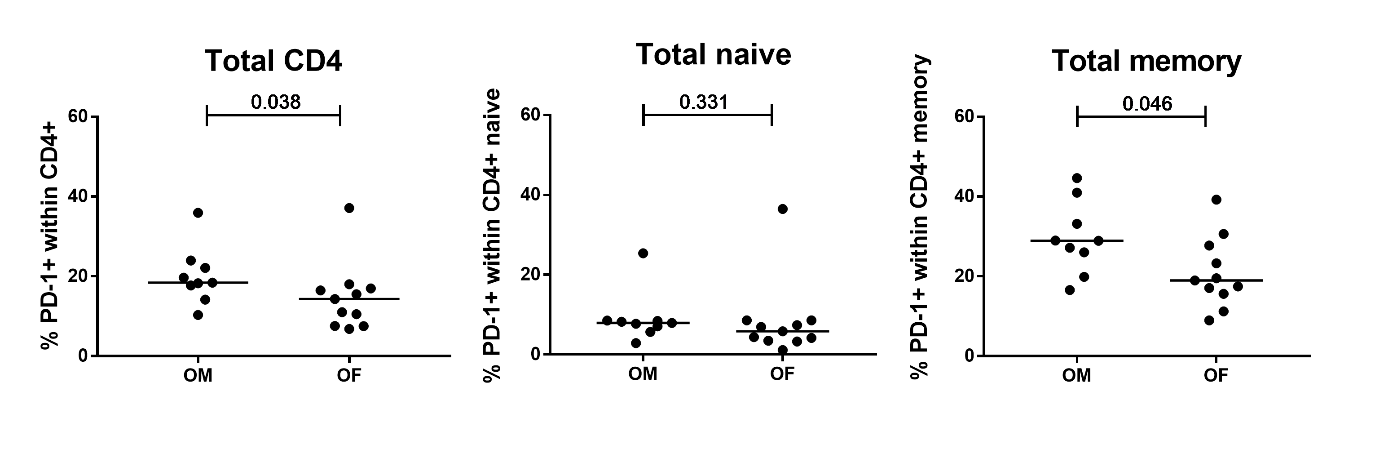


***Additional figure S8. Percentages of PD-1+ cells within total, naive and memory CD4+ T cells in older males and females.*** *Sex effects on PD-1 expression were determined by flow cytometric staining of whole blood. Horizontal bars reflect median percentages. The Mann-Whitney U test was used to compare frequencies of PD-1 cells between older males (OM, n=9) and older females (OF, n=11) adults. P-values are indicated in the graphs.*
